# Generating kidney tissue from pluripotent stem cells

**DOI:** 10.1038/cddiscovery.2016.53

**Published:** 2016-07-18

**Authors:** MH Little

**Affiliations:** 1 Kidney Development, Disease and Regeneration, Murdoch Childrens Research Institute, Flemington Rd, Melbourne, Victoria, Australia; 2 Department of Pediatrics, University of Melbourne, Melbourne, Victoria, Australia

## Abstract

With the isolation of human pluripotent stem cells came the possibility of generating specific cell types for regenerative medicine. This has required the development of protocols for directed differentiation into many distinct cell types. One of the more complicated tissue types to recreate is the kidney. Here we review recent progress towards the recreation of not only specific kidney cell types but complex kidney organoids, models of the developing human organ, *in vitro*. We will also discuss potential short and long term applications of these approaches.

## Study findings

- Directed differentiation of human pluripotent stem cells can generate cell types of all germ layers.
- Directed differentiation to kidney involves directing cell through primitive streak and intermediate mesoderm.
- hPSCs can be used to generate nephrons *in vitro*.
- hPSCs have also been directed to form complex multicellular kidney organoids.
- Such organoids have potential in disease modelling and drug screening.

**Figure 1:**
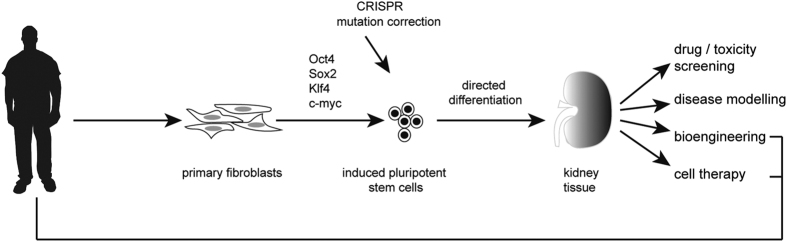
Potential applications for directing the differentiation of human pluripotent stem cells to kidney (adapted from Takasato *et al*.^25^).

A pluripotent stem cell is, by definition, capable of differentiating into all possible cell types. The pluripotent state within a developing embryo includes the inner cell mass of the preimplantation embryo and the subsequently derived epiblast. Although pluripotency in the embryo is regarded as a transient state, the isolation and continuous culture of such cells in a pluripotent state has been possible in mouse for more than three decades. The derivation of the first such human pluripotent stem cell line in 1998^1^ and the subsequent identification of key transcription factors able to convert an adult somatic cell into an equivalent pluripotent state,^2^ the induced pluripotent stem cell (iPSC), has totally transformed stem cell biology, opening the door for many regenerative medicine options. It is easy to understand how pivotal these findings have been given the fact that, in theory, you should now be able to make a renewable, expandable and patient-specific stem cell that can be directed to form the required cell type for treatment and deliver this back as an autologous treatment (Figure 1). This is the potential of iPSCs. In reality, there remain many obstacles, not least of all the ability to direct the differentiation of these stem cells to the cell type that is desired. Although early progress has been made with respect to *in vitro* directed differentiation of pluripotent stem cells to ectodermal end points, particularly specific neuronal subtypes, the generation of kidney cell types (derivatives of the intermediate mesoderm) has been slower to come. However, the last 2 years have seen substantial advances.

## Directing differentiation to kidney

**Figure 2:**
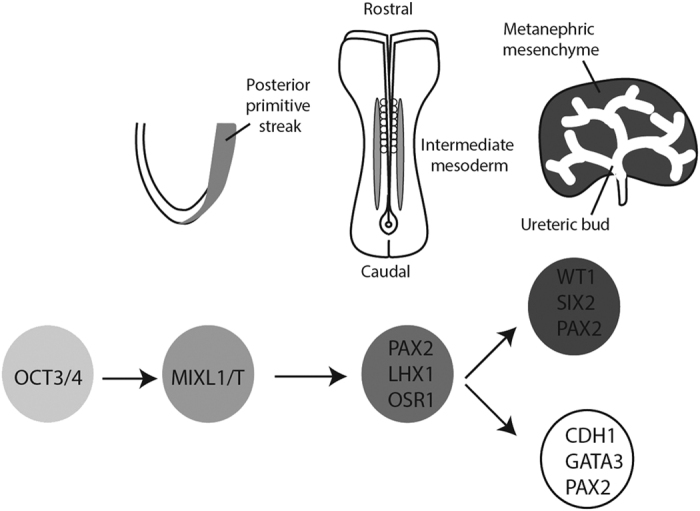
Embryological pathway for the development of the kidney, showing the critical differentiation milestones required for the formation of kidney tissue from stem cells. The genes serving as mileposts for directed differentiation of human pluripotent stem cells are outlined below each of the key stages of differentiation.

Like the muscles and the blood, the kidney is derived from the definitive mesoderm of the embryo.^3^ More specifically it arises, like the gonad, from the intermediate mesoderm and forms through interactions between two major cell types; an epithelial duct called the ureteric bud that forms the collecting ducts required for urine to leave the kidney and a metanephric mesenchyme, which gives rise to all the different cell types of the epithelial filtration units called nephrons^3,4^ (Figure 2). Our understanding of the formation of these cell types in other organisms, particularly the mouse, has guided protocols for the directed differentiation of human pluripotent stem cells to kidney cell types. Some of the earliest approaches to directing differentiation to kidney used a combination of growth factors known to be important either for early kidney formation or the specification of individual kidney cell types to look for differentiation into these cell types. In this way, groups have reported the formation of proximal tubular epithelium^5^ and glomerular podocytes^6^ from human embryonic stem cells. Other approaches have more systematically monitored the progression of the differentiating cells in culture through intermediate stages of development, including the primitive streak, intermediate mesoderm and into either the collecting duct epithelium^7^ or nephrogenic mesenchyme.^8–11^ Not surprisingly, most of these approaches have focussed on the addition of similar recombinant growth factors or small agonists, with an initial induction of primitive streak usually involving canonical Wnt signalling and/or activin/nodal and BMP signalling. This is followed by the addition of an FGF (either FGF2 or FGF9) and frequently the inclusion of BMP7 to support the formation of the nephrogenic mesenchyme. Some approaches have generated these tissues from monolayers of starting cells,^7,10^ whereas others have used embryoid body culture or even formation of an intermediate epiblast stage within matrigel.^9,12^ The convergence of differentiation protocols for the formation of early nephrons is striking in that a number of groups now show clear evidence for the formation of segmented and patterned nephrons with individual segments showing clear functional differentiation into proximal tubule, loop of Henle, distal tubule and the epithelial cell types of the glomeruli.^9–14^ Transplantation of such structures under the kidney capsule of a recipient animal have also been demonstrated to facilitate vascularisation of the glomeruli as would normally occur during nephron formation.^12,14^

## Formation of complex multicellular kidney organoids from human pluripotent cells

**Figure 3:**
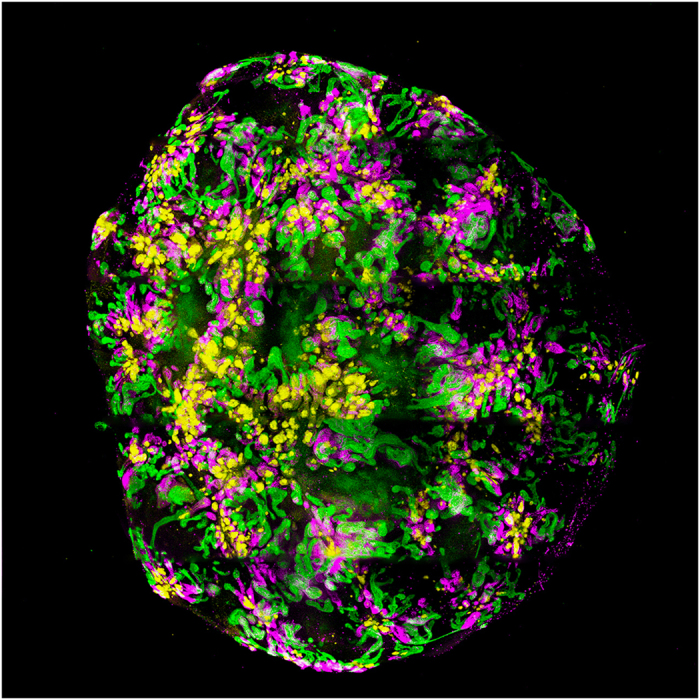
A kidney organoid generated from a human pluripotent stem cell line.^16^ This organoid has been cultured for 18 days as an aggregate post induction of intermediate mesoderm. Immunofluroescence displays the presence of differentiating nephrons comprised of glomeruli (NPHS1, yellow), proximal tubules (LTL, pink) and distal tubules/collecting ducts (CDH1, green). Image by Minoru Takasato.

**Figure 4:**
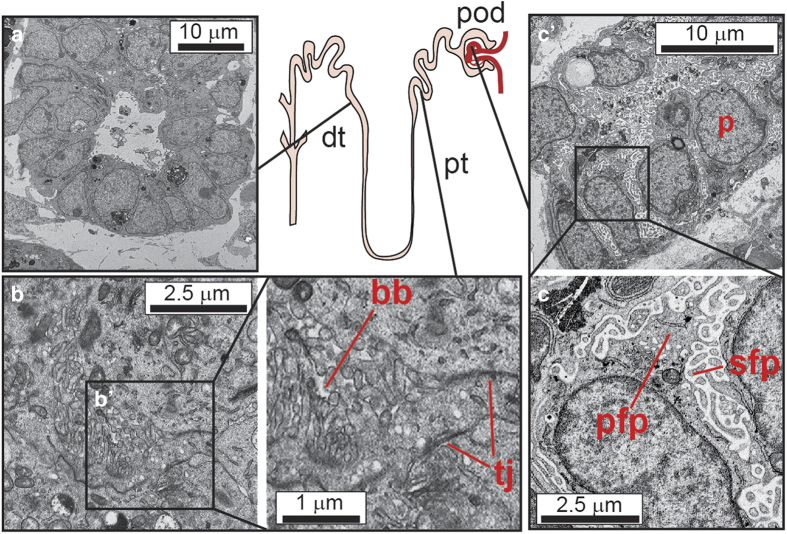
Transmission electron microscopy showing the presence of specific nephron cell types within the kidney organoids generated from human-induced pluripotent stem cells. A diagram of the presumed location of specific cell types along the nephron is shown centrally. (**a**) Distal tubular epithelium showing clear lumen and small apical microvilli (**b**, **bʹ**) Proximal epithelial tubules showing evidence (**bʹ**) of brush border (bb) and cell–cell tight junctions (tj) (**c**, **cʹ**) Forming glomerulus with evidence of tightly interdigitated podocytes (**cʹ**) with primary (fpf) and secondary (sfp) foot processes.

As differentiation of pluripotent stem cells often attempts to replicate development, it is not surprising to think that within a dish you might get multiple cellular outcomes rather than a single end point. This is, in fact, the case. Studies over the last few years have demonstrated the formation of complex multicellular organoids comprised of interacting component cell types arranged in an organotypic fashion. In this way, human pluripotent stem cells have been shown to form organoids of the developing eye (optic cup), the cerebral cortex, stomach and intestine (reviewed in Ader and Tanaka^15^). In each of these cases, the progenitors of the organ of interest self-organise in three dimensions as they might during normal development to form a model of the organ. We have recently demonstrated the formation of kidney organoids after the *in vitro* directed differentiation of human iPSCs cultured as a pellet at an air media interface^16^ (Figure 3). This is the same culture method that has long been applied to the *ex vivo* culture of mouse embryonic kidneys.^17^ Indeed, it is possible to completely dissociate an embryonic mouse kidney, reform an aggregate of the component cells and have the cells self-organise to reform the epithelial elements of the original organ.^18,19^ Presumably, therefore, the culture of the differentiated iPSC in this format facilitates a similar self-organising environment. Within these human kidney organoids, there is evidence of more than eight distinct cell types, including the formation of appropriately segmenting nephrons comprised of distal tubule, loop of Henle, proximal tubule and Bowman’s capsules containing parietal epithelial cells and podocytes (Figure 4). Simultaneously, the collecting duct epithelium forms and connects to the nephrons. Surrounding these epithelial elements, there is a stromal population that expresses key transcription factors known to mark the cortical stroma of the developing kidney, including Meis1. More surprisingly, an extensive endothelial capillary network arises, with an accompanying perivascular compartment. There is even evidence that a subset of the glomeruli begin to draw in these endothelial and pericytic progenitors to form the glomerular capillaries. The origin of the vasculature of the kidney has long been proposed to include both vasculogenic and angiogenic progenitors, however the origin of each of these progenitors types has been unclear. The presence of vasculature in such kidney organoids suggests that at least the vasculogenic component also arises from the intermediate mesoderm, as does the nephron progenitor population.^16^ The histological features of kidney organoids appear to represent relatively early kidney development. In agreement with this, an unbiased comparison of the expression profile of organoids with that of human foetal tissue most closely assigns kidneys' organoids to trimester one human kidney.^16^ What remains to be determined is how mature such *in vitro* organoids can become.

## Nephrotoxicity screening

With such progress in the generation of kidney cell types, the options for use of these cells have significantly widened (Figure 1). One early application has been the evaluation of iPSC-derived kidney cells for the screening of drugs to evaluate nephrotoxicity. The use of pre-clinical pharmaceutical exposure is currently the gold standard for nephrotoxicity screening. However, the mouse does not always predict the outcome in humans. The use of *in vitro* drug screening has been attempted using primary or immortalised proximal tubule epithelial cells, the principle cell type targeted by nephrotoxicants. However, accepted *in vivo* biomarkers do not appear to be induced in such screens and hence these are of low predictive value. The use of iPSC-derived proximal tubule cells has now been evaluated,^20^ with results suggesting greater accuracy in predicting toxicity than primary human proximal tubule cells.^20^ However, these were not evaluated for the induction of *Kim1*, the biomarker most widely regarded as an early and accurate predictor of nephrotoxic injury. With the generation of protocols for the creation of more complex kidney structures, including nephrons and whole organoids, has come early evidence that these respond to known nephrotoxicants via either specific proximal tubular apoptosis or increased production of Kim1 protein.^12,13,16^ Hence, these end points may also act as viable screens for nephrotoxic injury.

## Disease modelling using patient-derived stem cells

Although the majority of chronic kidney disease in adults is accepted as the consequence of accumulated insult, it is estimated that 50% of children reaching end-stage renal failure have an inherited form of kidney disease. The most common genetic cause of renal failure is autosomal dominant polycystic kidney disease (ADPKD), however there are many other heritable cystic kidney diseases (nephronophthisis and tubulointerstitial kidney disease), glomerulopathies and tubulopathies. Improvements in next generation sequencing over the last 5 years has led to the identification of many novel gene mutations in such conditions. However, as with many other diseases, there is significant variation in penetrance and expressivity within such families, likely due to accompanying variations in their genome. This makes the validation of any novel gene variant challenging. With the newly developed protocols for directed differentiation of iPSCs, it is now feasible to generate patient-specific lines for disease modelling and functional genomics (Figure 1). The capacity to scale up directed differentiation protocols may also facilitate patient-specific drug screening for the identification of new treatments. The first reports illustrating the proof of concept of kidney disease modelling have come with the demonstration of cystic epithelia within kidney tissue derived from an ADPKD iPSC line.^12^ There remain many hurdles to these studies. The identification of genuinely disease-specific changes in gene expression or developmental potential *in vitro* will need to be carefully distinguished from differences arising due to variations between individual iPSC clones and even between individual differentiation experiments (technical variation). It will also be incredibly important to ensure that any ‘control’ comparison is performed with lines from a closely related individual, preferably an isogenic clone from the patient themselves corrected for the mutation of interest. The advent of efficient and more accurate gene editing technologies, such as CRISPR, are making this possible.

## Options for regenerative medicine: future directions

Although the cultures of kidney cells, or even the more complex kidney organoids, is likely to deliver results around disease modelling and drug screening in the short term, the longer term goal of regenerative medicine is some way off. At present, kidney organoids reach ~8 mm in diameter after ~3 weeks in culture with each organoid containing up to 100 nephrons.^16^ Each human kidney contains, on average, 1 million nephrons.^4^ After renal failure, dialysis provides renal function equivalent to 10–15% of glomerular function, but even this suggests the need to generate a kidney replacement with >100 000 nephrons. The challenge does not end with scaling up nephron number, as the capacity of the kidney to appropriately reclaim fluids, amino acids, electrolytes and other nutrients requires a particular histological topology as well as an integrated collecting duct network with a viable exit path to the bladder. This has not yet been achieved. Hence, generating an entire functionally competent replacement organ remains a major challenge. One possible approach using iPSC-derived kidney cells might be the delivery of these cells back into decellularised scaffolds generated from human kidneys. Certainly, approaches for the de- and re-cellularisation of human kidney have been reported.^21^ It is possible that the generation of specific renal cell types for delivery back into the injured kidney – cellular therapy – may also provide some value. Early data suggests that this might be the case with the delivery of human-derived renal progenitors into mouse models of injury showing evidence of reduced damage,^22,23^ but it remains to be seen whether such cells can functionally integrate long term and whether they will do so in the face of chronic renal injury. What may be more feasible is the use of kidney cell types generated from iPSC in microfluidics-based organ-on-a-chip technology.^24^ Such options remain in the future. However, identifying the methodology to move a pluripotent stem cell state to a kidney end point has opened the door to all of these possibilities. It also opens the door, for the first time, to a better understanding of the molecular basis of normal kidney development in the human and it is here that there is much to be learned.
